# Hepatitis C and Why the Treatment is Needed Now? The Summary Report From the Cross-Border Symposium of the 5th Tehran Hepatitis Congress May 2013

**DOI:** 10.5812/hepatmon.16082

**Published:** 2013-11-20

**Authors:** Nasser Ebrahimi Daryani, Seyed Moayed Alavian, Mohammad Hossein Somi, Mohammad Torabi-Nami

**Affiliations:** 1 Department of Gastroenterology and Hepatology, Tehran University of Medical Sciences, Tehran, IR Iran; 2 Baqiyatallah Research Center for Gastroenterology and Liver Disease, Baqiyatallah University of Medical Sciences, Tehran, IR Iran; 3 Middle East Liver Disease Center (MELD), Tehran, IR Iran; 4 Research Center for Gastroenterology and Liver Disease, Tabriz University of Medical Sciences, Tabriz, IR Iran; 5 School of Advanced Medical Science and Technologies, Shiraz University of Medical Sciences, Shiraz, IR Iran

**Keywords:** Hepatitis C, Protease Inhibitors, Management, Iran

## Abstract

The cross-border symposium on hepatitis C, entitled “why treating now?” was held on 15th May 2013 during the 5th International Tehran Hepatitis Congress. The present report summarizing communicated insights during this symposium is intended to help health care providers to make well-informed decisions when treating patients with chronic hepatitis C (CHC). Since today’s evolving science of hepatitis C management has introduced new treatment options, one should be well-versed about the potential benefits as well as untoward effects or practical challenges when using these regimens. In addition to outline HCV treatment advances, this symposium focused on the central question that why eligible patients with hepatitis C who may mostly benefit from the currently available protease inhibitors, should be treated now rather than be waited for the future therapies. Moreover, an overview of long term local experience with protease inhibitors in our challenging hepatitis C patients was presented during this interactive symposium.

## 1. Introduction

The therapeutic strategies for chronic hepatitis C (CHC) has notably evolved over the past two decades. Treatment protocol began with interferon alpha (IFN-α) monotherapy in 1993, thereafter it moved on adding ribavirin (RBV) to IFN-α in 1998, and finally pegylated IFN-α (PegIFN-α) was emerged in 2000. The combination therapy with PegIFN-α and RBV became the standard of care since 2001(1, 2). Currently, despite the introduction of direct-acting antivirals (protease inhibitors or PIs) including Boceprevir and Telaprevir since 2011, and the ongoing research for new HCV therapies, PegIFN-α plus RBV have remained the backbone of HCV treatment (2, 3).

Our local experience with PegIFN-α plus RBV combination therapy over the past years has shown that 50 to 70% of the patients achieve the sustained virological response (SVR) depending on their genotype (whether 1a or non-1a), and other predictive variables (4-6). Nevertheless, given the lack of expected response or failed prior therapy in distinct category of patients (i.e. either naïve or treatment-experienced genotype 1 HCV-infected patients), the PI (Boceprevir or Telaprevir)-included triple therapy has become indicated (7-10).

The most recent international guidelines for the diagnosis and management of hepatitis C (11, 12), have recommended the PI-based triple therapy for patients with genotype 1, regardless of their prior treatment response. However, considering the cost and availability issue of such treatment regimens, local recommendations would assist clinicians with their decision-making in this regard. The recommendations laid down by the experts panel during the scientific leaders’ meeting, July 2011, Tehran (13), re-emphasized the significance of evidence-based decisions for using any new HCV therapy regimen in Iran; whereby, cost-benefit analysis should be carefully considered before decision making.

Given this, a clear understanding on these regimens’ implications, benefits, untoward effects or practical challenges are needed. This symposium tried to highlight: (1) why timely treatment with the currently available PI-included triple therapy is needed for a distinct category of patients, and waiting for future therapies is normally not recommended, (2) what practical considerations must be noted when applying these regimens, and (3) where we stand regarding our local experience with PI-included triple therapy for GT1 HCV-infected patients.

### 1.1. Today’s Landscape of Hepatitis C Treatment

When navigating the new landscape of hepatitis C treatment with the currently approved direct acting antivirals (DAAs), some questions emerge. Some fundamental issues which need to be clarified include: (1) which patients should be treated with these regimens? (2) what preparations are mandated before initiating the therapy? (3) how should we manage possible adverse events (AEs) ?, and (4) when the treatment should be stopped?.

According to the guidelines, patients with at least 18 years of age, having detectable genotype (GT) 1 HCV RNA in the serum, with a compensated liver disease, and liver biopsy showing a significant fibrosis (bridging fibrosis or higher) resemble the portrayal of cases in whom timely initiation of PI-included HCV treatment is usually not debated (11, 12).

Before commencing the HCV treatment, some clinical, hematological, and biochemical indices should be evaluated, and the baseline proper status needs to be ascertained. The absence of evidence favoring hepatic encephalopathy or ascites, total serum bilirubin of less than 1.5 gr/dL, international normalized ratio (INR) of less than 1.5, albumin > 3.4 gr/dL, and the platelet count of at least 75000/mm^3^ are amongst the crucial baseline requirements to start HCV therapy with the new DAA (protease inhibitors)-included regimens. Some further essential hematological as well as biological indices include hemoglobin (Hb) > 13 gr/dL for men, and >12 gr/dL for women, neutrophil count of more than 1500 cells/mm3, and the serum creatinine level of less than 1.5 mg/dL (7, 14-17).

Adding to the above, the two cardinal factors which motivate physicians to start HCV therapy with the newly available HCV treatment are patient’s willingness to treatment, and to conform to the treatment requirements as well as lack of treatment contraindications.

The natural course of HCV infection leaves over 80% of the afflicted cases to become chronic, of which almost 20% end up with cirrhosis within 10-20 years since diagnosis. Cirrhosis in turn leads to possible progression to the end-stage liver disease, and hepatocellular carcinoma (HCC) with an incidence rate of 6% per year, and 4% per year, respectively. The rate of liver transplantation and death is 3-4% per year when these complications develop (18). While decompensated cirrhosis is known to be a contraindication for triple therapy, compensated cirrhosis should not be excluded from such treatment regimens (19).

Given the potential post liver transplantation complications as well as the minimal (< 5%) five-year survival among patients with HCC, timely treatment of HCV to prevent disease progression into devastating liver damages, and the terminal disease state is believed to be a “must do” (2, 12).

Treatment is meanwhile contraindicated in pregnant patients and those contemplating pregnancy or unwilling to assure contraception. Since interferon (either pegylated or non-pegylated) and ribavirin (RBV) are pregnancy category X and C respectively, and protease inhibitors (Boceprevir or Telaprevir) are to be only used in combination with PegIFN and RBV, PIs become contraindicated in pregnant patients and couples planning pregnancy. Other contraindications for PI-included triple therapy are severe and uncontrolled concurrent diseases such as hypertension, heart failure, coronary artery disease, chronic obstructive pulmonary disease, poorly controlled diabetes mellitus, and known hypersensitivity to any of the drugs used in the triple combination (20-25).

Regarding HCV treatment, further advancements are already underway which have promised combination therapies purporting even more pronounced efficacy and less adverse events, as well as interferon-free regimens (26, 27).

Given the above, an almost universal question for patients with genotype-1 related CHC is whether they should be treated now of wait for future therapies. In reply to this question, current practice guidelines have left a large group of patients and treating physicians to individually weigh the risks against benefits of initiating therapy vs. watchful waiting (2, 11). Meanwhile, clinical evidence has suggested that patients with intractable CHC-related symptoms such as fatigue and those with extra hepatic manifestations including cryoglobulinemia, renal disease, and dermatologic manifestations should be treated now regardless of their stage of liver disease (11). Due to the poorly defined high progression rate of CHC, some other groups of patients are also recommended to be treated now. This subset of patients with initial high risk factors and faster progression of fibrosis include patients with infection after the age of 40, male gender, excessive alcohol consumption, HBV or HIV coinfection, steatosis, and prolonged immunosuppressed state (2).

Even in those CHC patients whose liver biopsy shows limited portal fibrosis (i.e. METAVIR score of 1 and 2), careful decision about the time of therapy should be made as in many instances treating now is better if there is no major burden or contraindication (11, 18).

In addition, in patients with high fibrosis level and comorbidities, waiting for future therapies leads to a missed window of opportunity to successfully eradicate the virus using the currently available therapy. Apparently, eradicating the virus not only provides benefits to the patients, but also decreases the risk of viral transmission to the community (2).

In fact, while the nascent field of HCV therapy with its evolving science has offered promise for interferon-free regimens (27-29), many issues remain unknown including the time to approval, worldwide availability, cost burden, safety profiles, and most importantly their impact on viral resistance as well as the durability of virological response. Consequently, we need to ascertain to what extent “waiting” is safe for patients when replying to their question “shall I be treated now?”(2).

## 2. Hepatitis C Treatment; A Changing Era

The combination regimen of PegIFN plus RBV has resulted in the sustained virological response (SVR) rate of almost 80% in GT2 and 3, and 45% in GT1. This regimen has remained the standard of care since 2001. Meanwhile, considering cellular and molecular pathways, which partly govern HCV replication and the response to IFN, in 2011, the direct acting antivirals (DAAs) as protease inhibitors (PIs) were added to PegIFN+RBV (R/R) regimen increasing the overall GT1 SVR rate up to 75% (7, 14, 30).

Since 2011, the international guidelines have put forward the PI-included triple therapy (i.e. Boceprevir or Telaprevir together with P/R) as the standard of care for GT1 HCV. These PIs directly interfere with the HCV polyprotein processing and viral protein function acting on a nonstructural (NS) serine protease known as NS3/4A (11, 12, 30, 31).

While we have recently started to experience the added value of the currently available PIs (Boceprevir and Telaprevir), there are some underway regimes expected to be available in coming years; however, their approval time is not clear yet. These molecules include Simeprevir, and Faldaprevir (to be added to P/R) for GT1 (28) as well as Sofosbuvir, (27) and Daclatasvir (32) for all genotypes. Sofosbuvir (as NS5B PI) is added to RBV and provides an IFN-free regimen for GT2 and 3 (27). The critical question of “why treatment with the currently available (Boceprevir or Telaprevir) PI-included therapy should be considered for many patients now, while for some others waiting for future therapies might possibly be considered” has been the focus of this report.

From the clinical point of view, factors predictive of a high likelihood of response to PI-based triple therapy are white race, low fibrosis level, rapid virological response (RVR), previous relapse following P/R therapy (vs. previous partial or null-response), and favorable IL28 CC genotype which is a marker of IFN responsiveness (33).

When determining HCV candidates for Boceprevir or Telaprevir, treating physicians should bear in mind the contraindications and precautions for using these agents in specific patients. In fact, all contraindications to P/R apply to these agents. Some contraindications include end-stage decompensated liver disease, coinfection with HIV (there are ongoing studies for HIV coinfections for BOC and TLV) or HBV, pediatrics, organ transplantation, and coadministration with other drugs which are highly dependent on CYP3A for clearance or strongly induce this cytochrome (24, 25).

### 2.1. Boceprevir-Included Triple Combination in HCV Treatment

**Figure 1.**
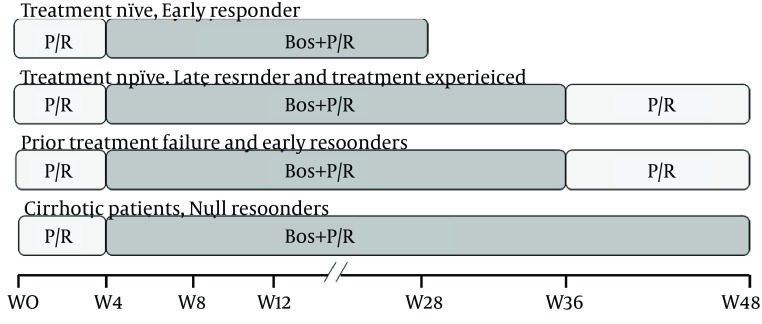
Boceprevir-Included Triple Combination and the Response Guided Therapy (RGT) The duration of BOC containing triple therapy depends on the initial treatment response at week 8 of therapy (week 4 of triple combination), and the virological response documented at week 24 for naive patients. As demonstrated in the diagram all treatment schedules possess a 4 week P/R (lead-in) treatment phase, and boceprevir is added at week 4. In treatment naive early responders (negative HCV RNA at week 8), the schedule would be 4w (P/R) + 24w (B/P/R). In treatment experienced patients and treatment naive late responders (HCV RNA detectable at week 8 and undetectable at week 24), also in nonresponders with negative results for HCV RNA at week 8, the treatment would include 4 w (P/R) + 32w (B/P/R) + 12w (P/R). Finally, in null-responders and patients with cirrhosis regardless of on-treatment or prior response, the schedule is 4w (P/R) + 44w (B/P/R). Boc: boceprevir, P/R: Pegylated Interferon α/Ribavirin.

Boceprevir is always used in combination with P/R to treat GT1 HCV. The triple combination comprises PegIFNα (2b: 1.5 µg/kg/wk or 2a: 180 µg/wk, subcutaneously) plus RBV (800-1400 mg/day) plus Boceprevir 200 mg cap (800mg TID, every 7-9 hours with a light meal or snack). The treatment schedule includes 4 weeks of P/R as the lead-in phase followed by Boceprevir-included triple therapy for which the response-guided therapy (RGT) applies for naive patients (Figure 1) ( 7 , 8 , 24 ). 

### 2.2. Telaprevir-Included Triple Combination in HCV Treatment

**Figure 2.**
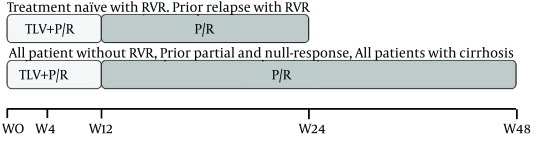
Telaprevir-Included Triple Therapy for GT1 HCV The duration of Telaprevir-included triple therapy is 12 weeks, and response-guided therapy (RGT) does not apply to this fixed treatment phase. However, the whole treatment duration (24 vs. 48 weeks) is determined by the achievement of the rapid virological response (RVR) which corresponds to un-detectability of HCV RNA 4 weeks after the initiation of Telaprevir-included triple therapy. TLV: Telaprevir, P/R: Pegylated Interferon α/Ribavirin.

Telaprevir should be administered in combination with P/R beginning on the first day. This triple combination is given for a fixed duration of 12 weeks after which the whole treatment duration with P/R (24 vs. 48 weeks) is determined based on the RVR achievement (Figure 2).

Each dose of Telaprevir is 750 mg (two 375mg tablets) which should be taken with approximately 20 g fat-containing food. The triple combination comprises PegIFNα (2b: 1.5 µg/kg/wk or 2a: 180 µg/wk, subcutaneously) plus RBV (800-1400 mg/day) plus Telaprevir 375 mg tab (750 mg TID, every 7-9 hours) ( 25 ). 

**Figure 3.**
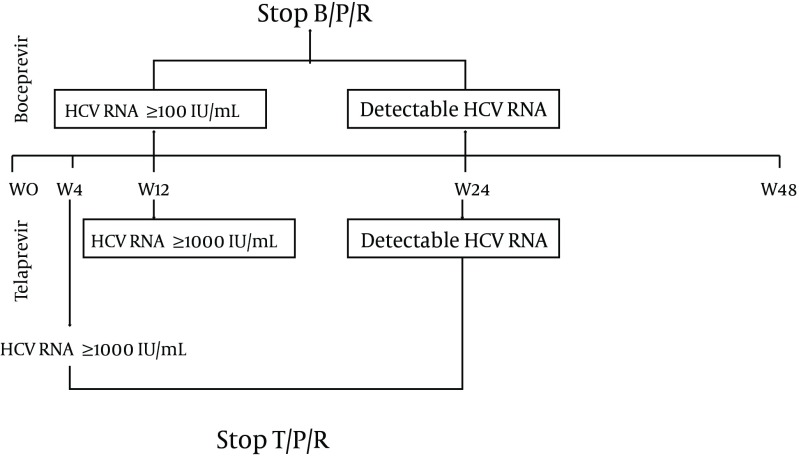
The Futility (stopping) Rule When Treating HCV With Boceprevir- or Telaprevir-Included Triple Therapy The futility rule which applies to Boceprevir is the HCV RNA≥ 100 IU/mL at week 12 or HCV RNA detectable (≥ 10-25 IU/mL) at week 24. The similar rule applies to Telaprevir; whereas, HCV RNA ≥ 1000 IU/mL at week 4 or week 12, and detectable HCV RNA (≥ 10-25 IU/mL) at week 24 mandate discontinuation of IFN, and hence the triple combination. B/P/R: Boceprevir + PegIFN + RBV, T/P/R: Telaprevir + PegIFN + RBV.

Depending on the HCV RNA level or its detectability at distinct milestones upon treatment with Boceprevir- or Telaprevir-included tripe therapy, the therapy is perceived non-efficacious, and hence should be stopped. The futility (stopping) rules for Boceprevir and Telaprevir slightly vary. This has been summarized in Figure 3. 

Neither Boceprevir nor Telaprevir should have dose modifications, and if either of the drugs is stopped for any reason it should not be restarted. Using either of these PIs is recommended to strictly follow their summaries of characteristics (24, 25).

### 2.3. Anticipated Untoward Effects With PIs

Both Boceprevir and Telaprevir may have potential and life-threatening side effects; thus, health care providers need to be well-informed and trained how to manage these possible adverse events. The most common side effects of Boceprevir in combination with P/R include fatigue, anemia, nausea, headache and dysgeusia; whereas, the most common side effects of Telaprevir in combination with P/R are rash, with or without pruritus, anorectal burning sensation, and anemia. Telaprevir-induced rash (occurring in almost 35% of cases) is usually mild; however, it can become severe, and even lead to hospitalization. A recent report has indicated the Telaprevir-included combination therapy-associated anemia to be 37% vs. 19% for P/R. This adverse event is reported to occur in 49% vs. 29% of Boceprevir-included combination therapy vs. P/R, respectively (24, 25, 34, 35).

Noting the benefits and challenges of the currently available PI-included regimens for HCV, well organized trainings are needed to provide practical skills to those who have considered using these regimens for their challenging-to-treat patients. For some specific patients i.e. naïve or treatment-experienced GT1 hepatitis C cases who are strongly willing to be treated with these regimens, patients with high stages of liver fibrosis, and those with comorbidities, the timely management of their HCV with these regimens (rather than waiting for possibly easier regimens in the future) is recommended (2, 12, 28). In addition, whomever we can treat now is one less patient we need to be worried about in the future. 

## 3. Local Experience With Protease Inhibitors; the Present Status in Iran

Following the first consensus meeting held in July 2011, Tehran, Iran, the preliminary agreements on the overall strategies for using PI-included regimens in challenging-to-treat hepatitis C patients were made (13). The expert attendees tried to draw a practical image to promote the use and prevent the misuse of these newly available regimens. Based on the agreed upon algorithms during the above consensus meeting (13), field experts started to shortlist their hard-to-treat cases ( mainly GT1 relapsers and nonresponders to the standard P/R regimen) to let them through a new promising treatment journey using PIs.

Consideration of these regimens rooted in the unmet needs with P/R. For instance, the highest success rate ( SVR achievement) using the standard P/R regimen in Iranian patients with thalassemia turned to be 51%; therefore, almost a half of these patients have been either nonresponders or relapsers (4).

According to a recent report (5), amongst GT1a-HCV patients (the most prevalent GT in Iran), 50% achieved SVR, 12% became nonresponders, 22% relapsed, and 16% experienced the breakthrough relapse. In a multivariate analysis, favorable predictive factors for SVR aged 40 or younger upon treatment, non-1a GT, and the baseline HCV RNA level of less than 600,000 IU/mL (5).

Published evidence has demonstrated an increase in SVR among nonresponders upon triple therapy with Boceprevir (SVR rate of up to 52%) (8). In relapsers, when Boceprevir is administered after a 4-week lead-in period, the rate of SVR is as high as 69%-75% (8). The lead-in phase allows for the real-time assessment of the patients’ response to P/R and helps assessment of the likelihood of SVR achievement. Furthermore, lead-in allows RBV to reach a steady state concentration thus may reduce the potential for resistance in patients responsive to P/R by decreasing the viral load. It also enables the assessment of patients’ adherence and tolerability before adding the PI (8, 36).

Besides, the optimal treatment outcome depends on the virus kinetics. Those patients who demonstrate undetectable HCV RNA at week 4 of the treatment ( following P/R) are more likely to achieve SVR than patients who have undetectable virus by week 12 (82%-94% vs. 79%) (7).

Evaluation of the liver disease status is recommended before the treatment strategy is defined. With respect to liver fibrosis, when the fibroscan reveals an advanced fibrosis (F3-F4 or F4), even naive GT1 patients may become candidate for a PI-included triple therapy (37, 38). However, the clinical decision depends on the lack of contraindications for these regimens, as well as tolerability and affordability (12, 39).

There are some characteristic factors which leave an impact on patients’ response rate. These include IL28B and core-70 mutation status of the virus. When IL28B genotype is TT and the core-70 mutation is non-Arginine, the decision to start PI-included triple therapy becomes warranted even in naive cases (11, 40).

In our local setting, we have started to use Boceprevir-included triple combination in hard-to-treat CHC cases over the past 6 months. Some Iranian hepatitis experts have shortlisted almost 40 cases to receive this regimen; however, 23 patients have already been started on therapy and the remaining patients are currently on the waiting list. Due to the lack of an integrated and unified data gathering system (patients are from various centers in different provinces), long term efficacy and safety data of these patients could only be partly contemplated. Fortunately, Iran hepatitis network (IHN) has recently initiated to unifying the evaluation and treatment paradigm in a data registry. This would make contemplation of all patients’ clinical, disease-related and treatment data possible under a project entitled “Iran Hepatitis-C Cohort”. The infrastructure for this registry has been provided and IHN would proceed to launch this cohort in a few months. Within our collaborative network, we are currently incorporating our patients’ baseline characteristics, predictive and prognostic marker profiles, disease-related particulars, the so far on-treatment safety, and efficacy data as well as the treatment results in the above database through which an analytical report is expected to be made.

Clinical evidence in global scope has reported that treatment with PIs possibly leads to adverse events in patients with GT1 HCV infection. Anemia is expected to occur in approximately 40-45% of patients, from whom 40% tend to require erythropoietin administration (24). This concern has recently been reported to almost identically occur in Telaprevir- and Boceprevir-included treatment of GT1 HCV-infected patients (25). PI-associated anemia can be managed either by erythropoietin administration or RBV dose reduction (15, 36, 41).

Nevertheless, our recent experience has substantiated that amongst Iranian patients, Boceprevir is well-tolerated and anemia is of less severity. Understanding the reason behind this observation is worth a focused investigation.

## 4. Conclusions

HCV treatment paradigm has dramatically evolved over the past 20 years. This dynamic field has recently offered new hopes for hard-to-treat patients with chronic hepatitis C following the introduction of PIs (Boceprevir and Telaprevir)-included regimens. While P/R remains the backbone of HCV therapy, Boceprevir- or Telaprevir-included triple therapy is being considered as the standard of care for either naive or treatment-experienced GT1 HCV-infected patients. Some emerging anti-HCV therapies are underway. These direct-acting antivirals are expected to provide IFN-free regimens.

The question of why a distinct subset of patients ( especially those who are willing to be treated with the PI-included regimens today, patients with comorbidities, and those with high liver fibrosis level) need to be treated today and waiting for the future therapies is not warranted, was extensively discussed during the present symposium.

Iran Hepatitis Network (IHN) will soon launch the “Iran Hepatitis-C Cohort” to systematically register and follow up with the already enrolled PI-treated patients, and those who would receive either of the PIs from now on. IHN is delighted to welcome collaborations in this project.
